# Long-Chain Acyl-Carnitines Interfere with Mitochondrial ATP Production Leading to Cardiac Dysfunction in Zebrafish

**DOI:** 10.3390/ijms22168468

**Published:** 2021-08-06

**Authors:** Deung-Dae Park, Bernd M. Gahr, Julia Krause, Wolfgang Rottbauer, Tanja Zeller, Steffen Just

**Affiliations:** 1 Molecular Cardiology, Department of Internal Medicine II, University of Ulm, 89081 Ulm, Germany; deung-dae.park@uniklinik-ulm.de (D.-D.P.); bernd.gahr@uniklinik-ulm.de (B.M.G.); wolfgang.rottbauer@uniklinik-ulm.de (W.R.); 2 Clinic of Cardiology, Medical University Center Hamburg-Eppendorf, University Heart and Vascular Center Hamburg, 20251 Hamburg, Germany; j.krause@uke.de (J.K.); t.zeller@uke.de (T.Z.)

**Keywords:** long-chain acylcarnitine, cardiovascular disease, mitochondria, zebrafish

## Abstract

In the human heart, the energy supplied by the production of ATP is predominately accomplished by ß-oxidation in mitochondria, using fatty acids (FAs) as the primary fuel. Long-chain acylcarnitines (LCACs) are intermediate forms of FA transport that are essential for FA delivery from the cytosol into mitochondria. Here, we analyzed the impact of the LCACs C18 and C18:1 on mitochondrial function and, subsequently, on heart functionality in the in vivo vertebrate model system of zebrafish (*Danio rerio*). Since LCACs are formed and metabolized in mitochondria, we assessed mitochondrial morphology, structure and density in C18- and C18:1-treated zebrafish and found no mitochondrial alterations compared to control-treated (short-chain acylcarnitine, C3) zebrafish embryos. However, mitochondrial function and subsequently ATP production was severely impaired in C18- and C18:1-treated zebrafish embryos. Furthermore, we found that C18 and C18:1 treatment of zebrafish embryos led to significantly impaired cardiac contractile function, accompanied by reduced heart rate and diminished atrial and ventricular fractional shortening, without interfering with cardiomyocyte differentiation, specification and growth. In summary, our findings provide insights into the direct role of long-chain acylcarnitines on vertebrate heart function by interfering with regular mitochondrial function and thereby energy allocation in cardiomyocytes.

## 1. Introduction

**Figure 1:**
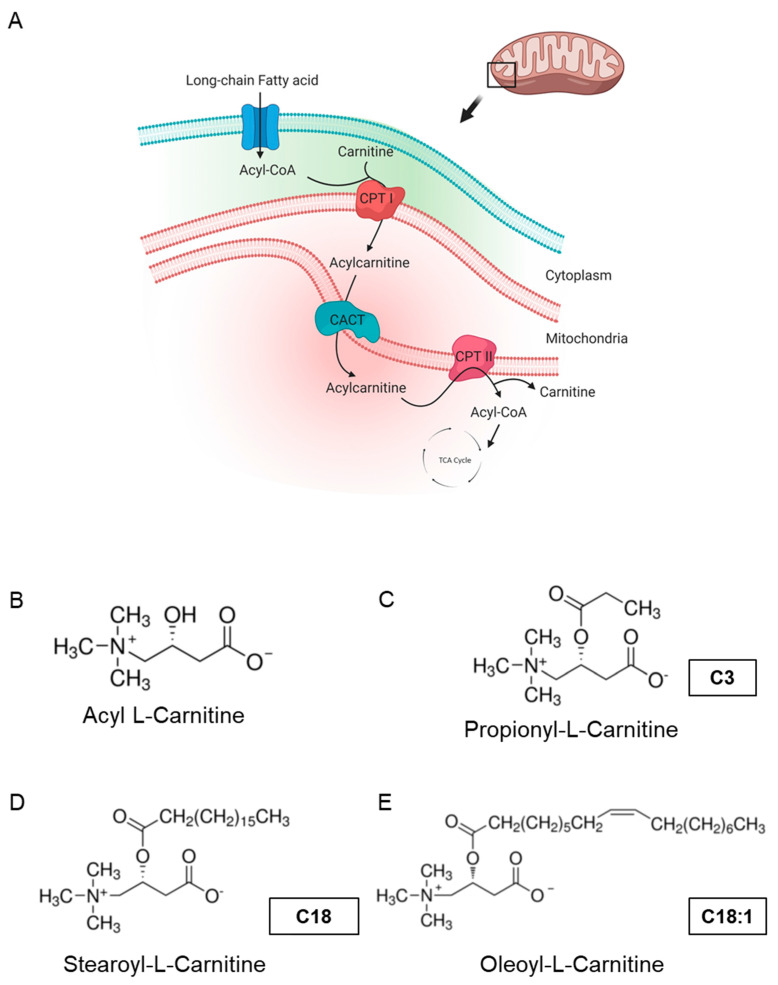
Carnitine shuttle in mitochondria and chemical structures of carnitines. (**A**) Description of acylcarnitine metabolism (free fatty acid metabolism) in general. Acyl-CoA and carnitine are conjugated (acylcarnitine) by carnitine-palmitoyl transferase I (CPT I) and transported across the inner membrane of mitochondria via carnitine-acylcarnitine translocase (CACT). Then, acyl-CoA and carnitine are released into the mitochondrial matrix by carnitine-palmitoyl transferase 2 (CPT II). Acyl-CoA is finally degraded and released as acetyl-CoA to enter the TCA cycle, synthesizing ATP. Subfigure A is created by BioRender.com (accessed on 23 March, 2021). (**B**–**E**) Molecular structures of acyl-l-carnitine, short-chain carnitine and long-chain acylcarnitines (LCACs).

Mitochondrial dysfunction has been implicated in the development of heart failure, which is the leading cause of hospitalization in all Western countries and is associated with high morbidity and mortality. Mitochondrial dysfunction often develops as the result of unsuccessful adaptation to energy stress in the heart, which perpetuates a maladaptive spiral, augmenting cardiac damage. In clinical findings, heart failure was found to be linked to defective carnitine transport and mitochondrial fatty acid (FA) oxidation [1]. Cardiac mitochondrial FA oxidation is a pivotal pathway for maintaining energy homeostasis and is a significant source of adenosine triphosphate (ATP) energy provision under restricted glucose accessibility. Due to an impermeable inner mitochondrial membrane, free long-chain FAs enter mitochondria via a specialized carnitine carrier system, transporting activated FAs from the cytosol to mitochondria (Figure 1A) and are enzymatically transformed into acyl-coenzyme A(-CoA) esters [2,3].

Acyl-l-carnitine (Figure 1B) belongs to the family of carnitines, a group of naturally occurring compounds that are essential for the β-oxidation of FAs in mitochondria during ATP synthesis [4]. Long-chain acylcarnitines (LCACs, e.g., C18 or C18:1) are intermediates in intramitochondrial FA metabolism. For instance, the long-chain acylcarnitine C18:1 is an intermediate transportation form for the monounsaturated FA C18:1 and high LCAC levels have been related to CVD, including heart failure (HF), coronary artery disease (CAD) and cardiac arrhythmias [5,6,7,8,9,10,11]. Hence, LCACs are not only potential CVD biomarkers, but might also be the putative cause of distinct cardiac disease.

The zebrafish has become a valuable vertebrate model in the study of human cardiac disease and has already successfully contributed to the identification and characterization of the genetic and molecular underpinnings of CVD, as well as the discovery and mechanistic description of bioactive small molecules. During the last decade, in vivo screening of small molecules using zebrafish has helped to gain insights into their pharmacological characteristics, such as absorption, distribution, metabolism, excretion and toxicity, which were limited in cell-based analyses [12]. Cardiovascular physiology is highly conserved between humans and zebrafish, which guarantees the meaningful translation of findings from the zebrafish model to the human situation [13,14]. Vice versa, numerous human cardiovascular drugs and biologically active small molecules have already been shown to mediate identical effects on zebrafish physiology [15,16,17,18,19,20].

Here, to evaluate the putative impact of the LCACs C18 and C18:1 on mitochondrial and cardiac function in the in vivo vertebrate model of zebrafish, we treated wild-type zebrafish embryos with C18 and C18:1 and found that treatment with both LCACs resulted in a significantly reduced expression of genes involved in mitochondrial function and ATP production, eventually leading to decreased ATP levels. Similarly to mitochondrial morphology, structure and density in cardiomyocytes, cardiac chamber differentiation, specification and growth were not affected by LCAC treatment. Finally, we found that LCACs led to severe heart failure phenotypes, accompanied by reduced heart rate and diminished fractional shortening of the cardiac chambers in C18- and C18:1-treated zebrafish embryos. Our findings imply that LCACs are a potential novel contributor to CVDs by regulating ATP production and thereby regulating the energy supply in cardiomyocytes.

## 2. Results

### 2.1. LCAC Treatment Interferes with Mitochondrial Function, Resulting in Diminished ATP Production

**Figure 2:**
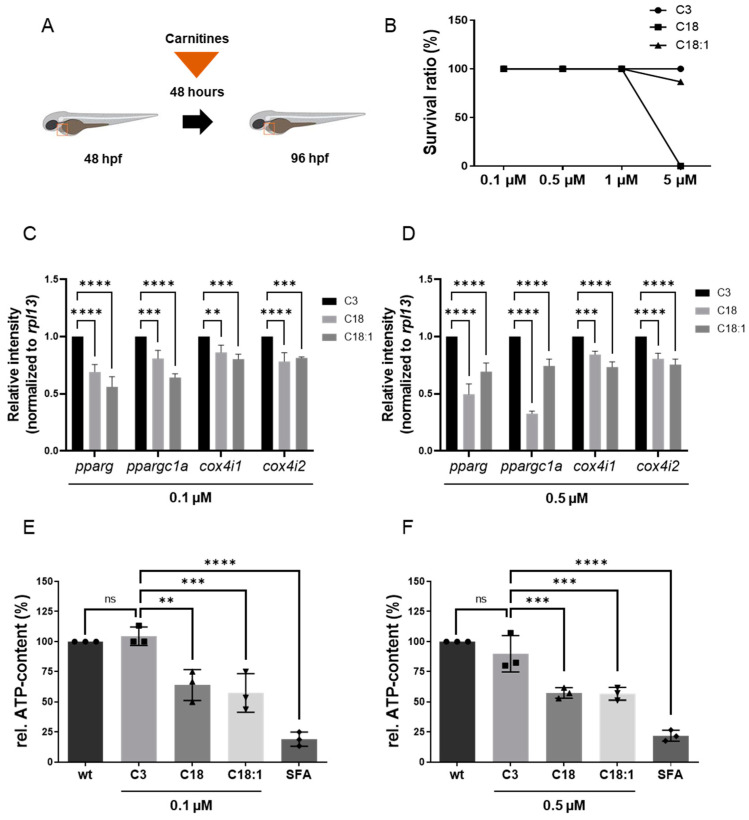
Experimental design of LCAC treatments in zebrafish embryos and survival rates for different concentrations of LCACs. Interrupted mitochondrial function by LCACs is accompanied by decreased ATP production. (**A**) Experimental design of compound treatments. (**B**) Lethality of the compounds at different concentrations. (**C**,**D**) Quantitative real-time PCR targeting genes related to ATP production (SD, *n* = 3, ns: *p* > 0.05, ** *p* < 0.01, *** *p* < 0.001, **** *p* < 0.0001). (**E**,**F**) Assessment of relative ATP content in zebrafish embryos after compound treatments (SD, *n* = 3, ns: *p* > 0.05, ** *p* < 0.01, *** *p* < 0.001, **** *p* < 0.0001). Abbreviations: rel. = relative, ns = not significant, wt = wild-type, SFA = sodium fluoroacetate.

**Figure 3:**
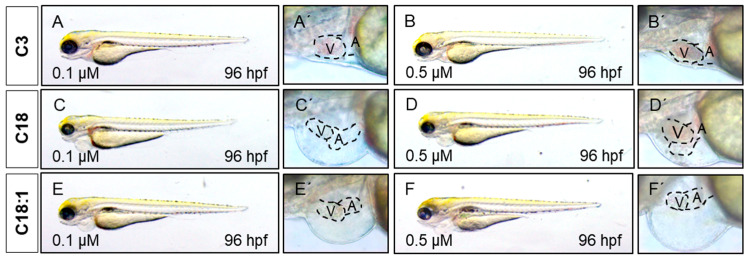
LCAC treatment induces cardiac phenotype in embryonic zebrafish. (**A**–**F**) Lateral view of embryos after 48 h of incubation with compounds. (**A**′–**F**′) Magnified view of hearts from subfigures **A**–**F**.

To assess the impact of long-chain acylcarnitines (LCACs) in vivo, we treated zebrafish embryos with the two LCACs C18 (stearoyl-l-carnitine) and C18:1 (oleoyl-l-carnitine), as well as the short-chain acylcarnitine (SCAC; propionyl-l-carnitine) C3 as a control (Figure 1C–E). First, embryos were incubated with 0.1, 0.5, 1 and 5 µM of C18, C18:1 or C3 from 48 h post-fertilization (hpf) for 48 h, to investigate potential adverse or toxic effects of the three molecules on zebrafish development, morphogenesis and survival (Figure 2A). By treating embryos with the different compounds, we found that 5 µM of C18 resulted in significantly increased mortality of zebrafish embryos, whereas 5 µM of C18:1 and C3 had no negative effects on embryo survival (Figure 2B). As we observed phenotypic changes such as pericardial edema and intracardiac blood congestion even with low concentrations (0.1 and 0.5 µM of LCACs) (Figure 3A–F and Supplementary Figure S1A–E), we decided to continue using these two concentrations in order to reduce the non-specific toxicity as much as possible. Nevertheless, these data persuaded us to exclude the high concentration from further analyses.

It is well known that LCACs (e.g., C14: myristoyl-L-carnitine, and C16: palmitoyl-L-carnitine) can suppress mitochondrial function by influencing Ca^2+^ homeostasis, leading to an inhibition of the TCA cycle and finally to the depletion of ATP [21]. Therefore, we evaluated whether C18 and C18:1 LCAC treatment of zebrafish embryos has an impact on mitochondrial function and subsequently the production of ATP. We first assessed the expression of marker genes such as *pparg*, *ppargc1a*, *cox4i1* and *cox4i2* (Supplementary Table S1), which are known to be downregulated in functionally compromised mitochondria [22,23]. We found that *pparg* and *ppargc1a* transcripts were indeed significantly decreased in C18- (0.1 μM: *pparg*: 0.69 ± 0.07, *ppargc1a*: 0.81 ± 0.07; 0.5 μM: *pparg*: 0.50 ± 0.09, *ppargc1a*: 0.33 ± 0.02, SD, *n* = 3, *p* < 0.001, *p* < 0.0001) and C18:1-treated embryos (0.1 μM: *pparg*: 0.56 ± 0.09, *ppargc1a*: 0.64 ± 0.03; 0.5 μM: *pparg*: 0.69 ± 0.08, *ppargc1a*: 0.74 ± 0.06, SD, *n* = 3, *p* < 0.0001) compared to C3-treated embryos (Figure 2C,D). Consistently, mRNA levels of *cox4i1* and *cox4i2*, which encode subunits of cytochrome oxidase c, involved in the mitochondrial respiratory chain, were also significantly downregulated after C18 (0.1 μM: *cox4i1*: 0.86 ± 0.06, *cox4i2*: 0.78 ± 0.08; 0.5 μM: *cox4i1*: 0.84 ± 0.03, *cox4i2*: 0.81 ± 0.05, SD, *n* = 3, *p* < 0.01, *p* < 0.001, *p* < 0.0001) or C18:1 (0.1 μM: *cox4i1*: 0.80 ± 0.04, *cox4i2*: 0.81 ± 0.01; 0.5 μM: *cox4i1*: 0.73 ± 0.05, *cox4i2*: 0.76 ± 0.05, SD, *n* = 3, *p* < 0.001, *p* < 0.0001) treatment compared to the C3-controls (Figure 2C,D).

To prove that impaired mitochondrial activity caused by LCAC treatment subsequently led to the reduction of ATP, we quantified ATP levels in C18-, C18:1- and C3-treated zebrafish embryos. As a control, we treated zebrafish embryos with sodium fluoroacetate (SFA), a known inhibitor of the TCA cycle [19]. We found a significant reduction of ATP levels in C18- (0.1 μM: 63.89% ± 12.73%; 0.5 μM: 57.25% ± 4.36%, SD, *n* = 3, *p* < 0.01, *p* < 0.001), C18:1- (0.1 μM: 57.36% ± 16.01%; 0.5 μM: 56.66% ± 5.26%, SD, *n* = 3, *p* < 0.001) and SFA-treated zebrafish embryos, whereas treatment of zebrafish embryos with C3 (0.1 μM: 104.44% ± 7.70%; 0.5 μM: 89.83% ± 15.04%, SD, *n* = 3) had no effect on ATP production (Figure 2E,F).

### 2.2. Mitochondrial Structure in Cardiomyocytes Is Not Impaired by LCAC Treatment

**Figure 4:**
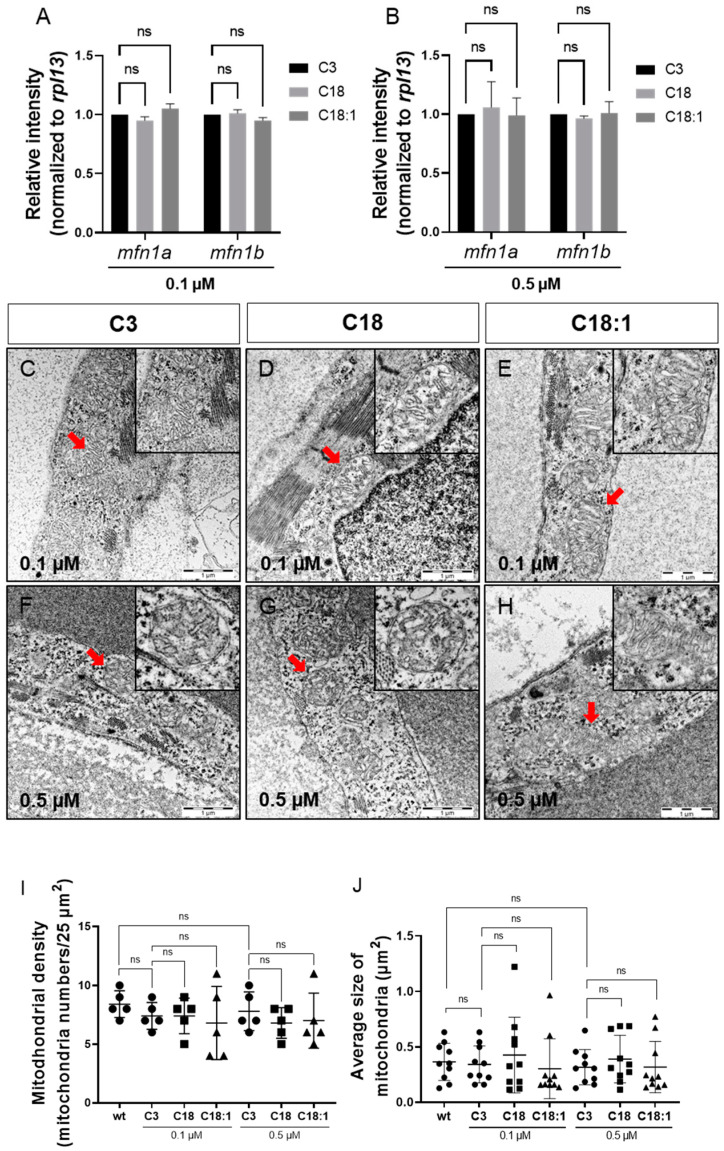
Sustained mitochondrial structure and distribution. (**A**,**B**) Quantitative real-time PCR targeting factors responsible for mitochondrial structure (SD, *n* = 3, ns: *p* > 0.05). (**C**–**H**) Mitochondria of compound-treated embryonic hearts visualized by means of electron microscopy. (**I**,**J**) Mitochondrial density and average size of mitochondria in EM images of embryonic zebrafish hearts treated with carnitines (SD, *n* = 5, 10: respectively, ns: *p* > 0.05). Abbreviations: ns = not significant, wt = wild-type.

To determine whether high LCAC uptake in mitochondria affects mitochondrial morphology and structure, thereby leading to defective ATP production, we first conducted quantitative real-time PCR (qRT-PCR) analyses to investigate the expression of *mfn1*, encoding the mitochondrial membrane protein involved in regulating mitochondrial morphology and metabolism [24,25]. We found the *mfn1a* and *mfn1b* mRNA levels in C18- and C18:1-treated embryos to be unaltered compared to the C3 control (Figure 4A,B and Supplementary Table S1). Next, we analyzed the mitochondrial ultrastructure in the ventricular and atrial cardiomyocytes of C3-, C18- and C18:1-treated embryos via transmission electron microscopy (TEM). We found that the mitochondrial structure in cardiomyocytes was not altered in C18- and C18:1-treated zebrafish embryos compared to control-treated (C3) or wild-type embryos (Figure 4C–H and Supplementary Figure S2A). The structure of the inner and outer mitochondrial membrane, as well as the cristae, were completely unaffected by LCAC treatment. Additionally, we found no other subcellular alterations in LCAC-treated embryos compared to C3 controls. Moreover, the mitochondrial density in C18- (0.1 μM: 7.4 ± 1.52/25 µm^2^, 0.5 μM: 6.8 ± 1.3/25 µm^2^, *n* = 5) and C18:1-treated embryonic hearts (0.1 μM: 6.8 ± 3.11/25 µm^2^, 0.5 μM: 7.0 ± 2.35/25 µm^2^, *n* = 5) was not significantly altered compared to C3-treated (0.1 μM: 7.4 ± 1.14/25 µm^2^, 0.5 μM: 7.8 ± 1.64/25 µm^2^, *n* = 5) or wild-type embryos (8.4 ± 1,14/25 µm^2^, *n* = 5) (Figure 4I). Next, we assessed mitochondrial size in C18-, C18:1- and C3-treated cardiomyocytes and found that the average size of cardiac mitochondria was also not impaired by C18 (0.1 μM: 0.43 ± 0.34 µm^2^, 0.5 μM: 0.30 ± 0.27 µm^2^, *n* = 10) or C18:1 treatment (0.1 μM: 0.30 ± 0.27 µm^2^, 0.5 μM: 0.39 ± 0.22 µm^2^, *n* = 10) compared to C3-treated or wild-type embryos (C3: 0.1 μM: 0.34 ± 0.17 µm^2^, 0.5 μM: 0.32 ± 0.16 µm^2^; wt: 0.37 ± 0.17 µm^2^, *n* = 10) (Figure 4J). These findings demonstrate that mitochondria density, morphology and structure are not impaired in cardiomyocytes treated with 0. 1 μM or 0.5 μM of LCACs.

### 2.3. Treatment of Embryos with LCACs Leads to Cardiac Dysfunction

**Figure 5:**
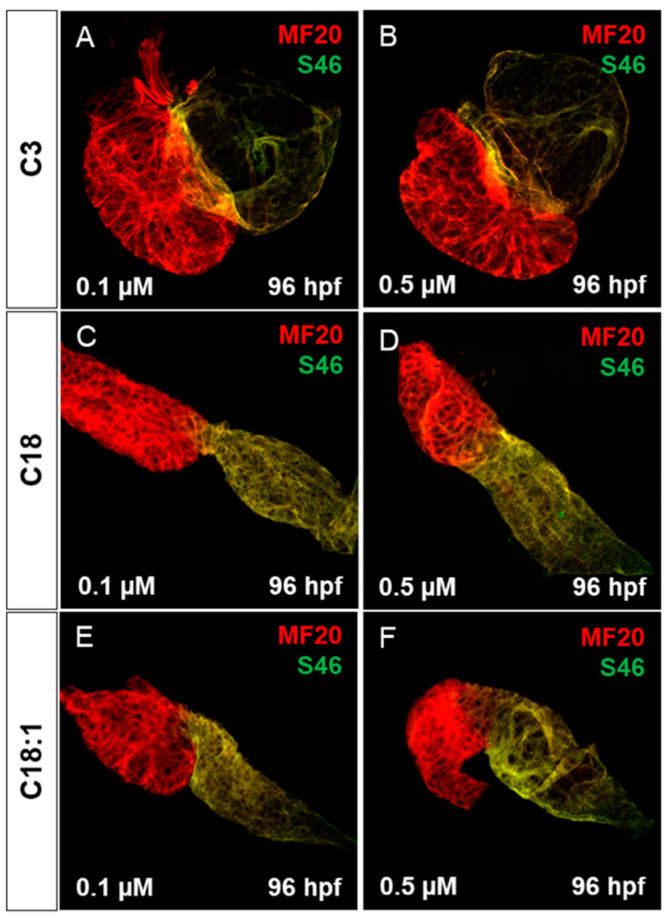
Cardiac chamber specification and development of LCAC-exposed zebrafish larvae. (**A**–**F**) Immunofluorescence staining of carnitine-treated embryonic hearts against sarcomeric myosin heavy chain (MF20) and slow developmental myosin heavy chain (S46).

**Figure 6:**
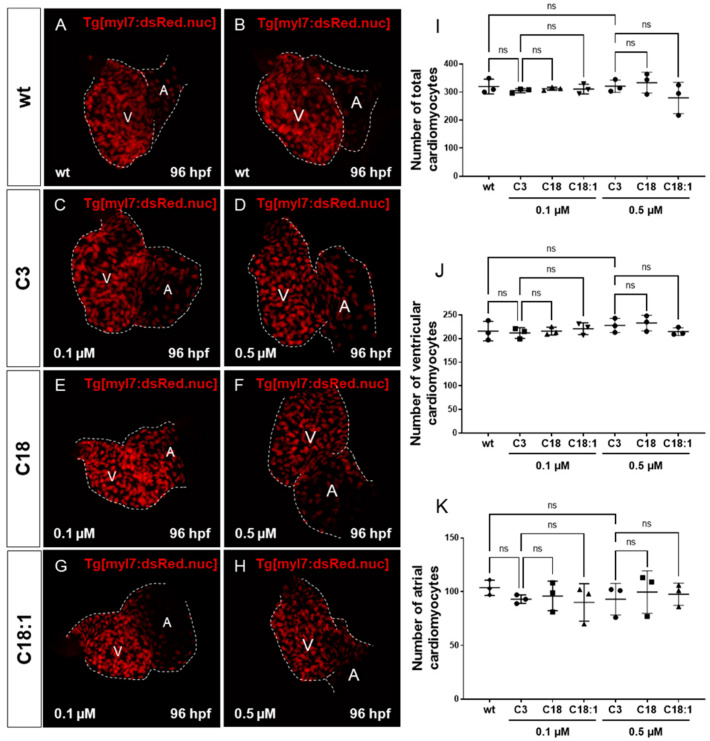
Impact of LCACs on cardiomyocyte numbers in zebrafish embryos. (**A**–**H**) Red-fluorescence-positive cardiomyocytes (CMs) of zebrafish embryos in wild-type and 0.1 M or 0.5 µM carnitine-treated embryos. (**I**–**K**) Counting CMs of whole heart, ventricle and atrium from wt and C3-, C18- and C18:1-treated embryos (total CMs: wt: 319.33 ± 26.08, C3 0.1 µM: 304.67 ± 7.57, C18 0.1 µM: 311.67 ± 5.51, C18:1 0.1 µM: 310.67 ± 17.56, C3 0.5 µM: 320.67 ± 21.78, C18 0.5 µM: 333.33 ± 37.17, C18:1 0.5 µM: 279.0 ± 55.75, SD, *n* = 3, ns: *p* > 0.05; ventricular CMs: wt: 215.67 ± 20.74, C3 0.1 µM: 211.67 ± 11.37, C18 0.1 µM: 215.67 ± 8.33, C18:1 0.1 µM: 220.67 ± 12.34, C3 0.5 µM: 227.67 ± 15.01, C18 0.5 µM: 232.67 ± 16.62, C18:1 0.5 µM: 214.67 ± 8.15, SD, *n* = 3, ns: *p* > 0.05; atrial CMs: wt: 103.67 ± 7.02, C3 0.1 µM: 93.0 ± 4.0, C18 0.1 µM: 96.0 ± 13.75, C18:1 0.1 µM: 90.0 ± 17.44, C3 0.5 µM: 93.0 ± 14.73, C18 0.5 µM: 99.67 ± 19.73, C18:1 0.5 µM: 97.67 ± 10.41, SD, *n* = 3, ns: *p* > 0.05). Abbreviations: ns = not significant, wt =wild-type.

It has been reported that mitochondrial dysfunction induced by specific mutations [19] or the pharmacological inhibition of mitochondrial activity [26] induces severe defects in the embryonic zebrafish heart. Since we observed mitochondrial dysfunction in C18- and C18:1-treated zebrafish, as well as a cardiac phenotype, we decided to characterize this phenotype in greater detail. The phenotype, characterized by a pericardial edema, was not observed in control-treated (C3-treated) embryos (Figure 3A–F,A’–F’). Pericardial edema in zebrafish embryos is a sign of either defective heart development or cardiac malfunction [19]. First, to evaluate whether the observed cardiac defects in LCAC-treated embryos were caused by impaired heart chamber differentiation and specification, we dissected embryonic zebrafish hearts at 96 hpf and subsequently performed immunostainings using antibodies directed against meromyosin (MF20) and the atrial-specific myosin heavy chain (S46) to visualize distinguishable ventricle and atrium features. As shown in Figure 5, similarly to the situation in embryos incubated with the control C3, we found that cardiac chamber differentiation and specification was completely unaffected in C18- and C18:1-treated zebrafish embryos (Figure 5A–F and Supplementary Figure S2B). Furthermore, to investigate whether LCAC treatment resulted in diminished cardiomyocyte numbers, we assessed total cardiomyocyte numbers and also counted the ventricular and atrial cardiomyocytes after treatments with C3, C18 and C18:1 separately. To do so, we treated embryos of transgenic zebrafish Tg(*myl7:dsRed.nuc*) expressing red fluorescence specifically in cardiomyocyte nuclei, with 0.1 μM and 0.5 μM of C18, C18:1 and C3 (Figure 6A–H) enabling the counting of cardiomyocytes, and found that neither the total cardiomyocyte numbers nor ventricular or atrial cardiomyocytes were reduced after treatment of the transgenic zebrafish embryos with 0.1 μM and 0.5 μM of C18, C18:1 or C3, or in the wild-type embryos (Figure 6I–K).

### 2.4. Long-Chain Acylcarnitine Treatment Impairs Cardiac Contractile Function

**Figure 7:**
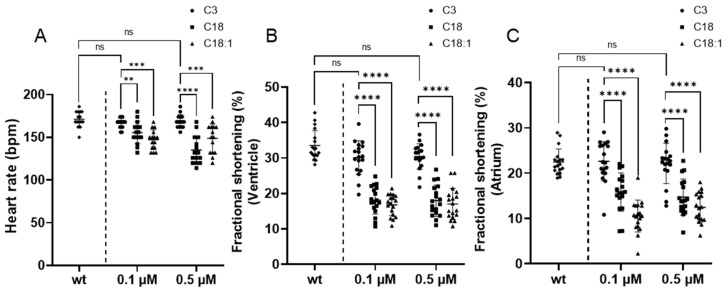
Cardiac dysfunction of zebrafish embryonic hearts resulting from LCAC treatment. (**A**) Comparison of heart rates between wt and compound-treated larvae. (**B**,**C**) Contractility of heart chambers (ventricle and atrium) after compound treatments compared to wt (SD, *n* = 15, ns: *p* > 0.05, ** *p* < 0.01, *** *p* < 0.001, **** *p* < 0.0001). Abbreviations: ns = not significant, wt = wild-type.

Since cardiac development was unaffected by LCACs treatment, we next assessed cardiac contractile function through the measurement of heart rate, as well as ventricular and atrial fractional shortening. The heart rate of C18- and C18:1-treated embryos was significantly reduced (C18: 0.1 μM: 150.13 ± 9.52, 0.5 μM: 136.07 ± 16.26; C18:1: 0.1 μM: 147.2 ± 11.53, 0.5 μM: 148.80 ± 16.85, beats per minute (bpm), SD, *n* = 15, *p* < 0.01, *p* < 0.001, *p* < 0.0001) compared to wild-type embryos (wt: 171.2 ± 9.31, bpm, SD, *n* = 15) and C3-treated embryos (C3: 0.1 μM: 166.4 ± 5.77, 0.5 μM: 168.8 ± 7.81, bpm, SD, *n* = 15) (Figure 7A). In addition, ventricular fractional shortening was also significantly compromised in C18- (0.1 μM: 18.11% ± 3.94%, 0.5 μM: 17.96% ± 4.38%, SD, *n* = 18, *p* < 0.0001) and C18:1-treated (0.1 μM: 16.77% ± 3.02%, 0.5 μM: 17.03% ± 4.41%, SD, *n* = 18, *p* < 0.001, *p* < 0.0001) fish compared to C3-treated (0.1 μM: 30.1% ± 4.75%, 0.5 μM: 30.45% ± 3.60 %, SD, *n* = 18) and wild-type fish (33.52% ± 4.22%, SD, *n* = 18) (Figure 7B). In addition to ventricular pump function, atrial fractional shortening was also decreased in C18- (0.1 μM: 15.83% ± 4.24%, 0.5 μM: 14.73% ± 3.99%, SD, *n* = 18, *p* < 0.0001) and C18:1-treated embryos (0.1 μM: 10.54% ± 3.53%, 0.5 μM: 12.44% ± 3.26%, SD, *n* = 18, *p* < 0.0001), whereas control-treated embryos were completely unaffected (C3: 0.1 μM: 22.63% ± 4.36%, 0.5 μM: 22.13% ± 4.44%; wt: 22.46% ± 2.88%, SD, *n* = 18), demonstrating that the LCACs C18 and C18:1 interfere with proper cardiac contractile function in vivo (Figure 7C).

These findings imply that the LCACs C18 and C18:1, but not the SCAC C3, interfere with mitochondrial function, leading to a depletion of ATP levels and finally resulting in a loss of regular heart function without affecting cardiac chamber differentiation or specification or cardiomyocyte numbers in vivo.

## 3. Discussion

The vertebrate heart is an organ with a very high energy demand. In this context, cardiac mitochondria are the predominant energy production plants and the dysfunction of mitochondria is linked to the development and progression of cardiac contractile defects and heart failure. Under physiological conditions, fatty acid (FA) oxidation is the main source of energy production in the myocardium. Metabolomic studies recently showed an association of high circulating levels of the FA intermediate long-chain acylcarnitine (LCAC) with cardiovascular disease (CVD) [9,27,28,29]. Here, LCAC plasma levels were correlated to the stage and severity of the disease, whereas a putative direct impact of LCACs on cardiac contractile function in vivo is still unknown. To assess the impact of LCACs on heart function in vivo, we treated zebrafish embryos at 48 hpf with C18 (stearoyl-l-carnitine) and C18:1 (oleoyl-l-carnitine). In accordance with the important role of LCACs in the ATP production of mitochondria, we observed significantly reduced mitochondrial function and ATP levels in treated embryos, whereas the morphology, structure and density of cardiac mitochondria were unaffected by excess C18 and C18:1 treatment. Furthermore, we observed the functional impairment of the embryonic zebrafish heart by measuring heart rate and fractional shortening, with significantly diminished cardiac function but without any effects on heart development and growth, suggesting that an insufficient energy supply accounts for the reduced heart rate and diminished fractional shortening in C18- and C18:1-treated zebrafish.

In the healthy human heart, the rate of energy supplied by ATP synthesis has to constantly match the rate of ATP consumption to perpetuate proper cardiac function. In this context, ATP production in the human heart is predominately accomplished via oxidative metabolism in mitochondria using FA as the primary fuel [30,31]. It was shown that the disturbance of the homeostasis between the energy supply and demand results in severe stress on myocardial energetics and eventually heart failure. The hypothesis that a failing heart is energy-starved is consistent with such a scenario and has inspired decades of research [32,33,34]. The downregulation of pathways involved in FA oxidation and the accumulation of incompletely oxidized FAs were observed in an early stage of heart failure, suggesting a mismatch between FA supply and oxidation [27]. On the other hand, promoting FA usage has been shown to be beneficial in a number of heart failure models [35,36], underlining the importance of orchestrated FA metabolism to guarantee regular cardiac function. In addition to ATP production through FA oxidation, glycolysis and phosphotransferase reactions are involved in energy production in the context of high energy demands, as is seen in failing hearts [37]. Therefore, the metabolism in cardiomyocytes is constantly remodeled. When the maximum capacity of ATP production is reached, or remodeling fails due to the loss of one or more components of the energy production system, ATP levels drop, leading to a functionally-important energetic deficiency, ultimately resulting in impaired contractile function [32,38].

Long carbon chains linked to FAs transform them into the most effective substrate for oxidative energy provision in the adult human heart. Interestingly, the clinical presentation of disturbed long-chain FA oxidation is quite unspecific, but one common feature in long-chain FA oxidation disorders is the fact that they can be provoked or aggravated by energy-requiring states. The most common clinical presentations in long-chain FA oxidation disorders are hypoketotic hypoglycemia, cardiomyopathy and myopathy occurring in combination or in isolation [3].

Long-chain acylcarnitines, the transport form of cytosolic long-chain FAs into mitochondria, play critical roles in cardiac metabolism and the supply of energy since they are the predominant source of ATP production in cardiac mitochondria via β-oxidation [31]. Recently, LCACs were described as blood-circulating biomarkers associated with cardiovascular diseases [39,40,41]. However, whether LCACs directly affect myocardial function, and particularly whether this effect is positively or negatively inotropic, is undecided [42]. Here, we treated zebrafish embryos with the LCACs C18 and C18:1 and observed significantly impaired mitochondrial ATP production, resulting in cardiac contractile dysfunction in treated embryos. Particularly, we found significantly decreased heart rates in embryos treated with C18 or C18:1, similar to the situation in the zebrafish mutant *schneckentempo*, which also showed a lack of ATP production due to a mutation in the *dihydrolipoyl succinyltransferase* (*dlst*) gene, known to be essential for the functioning of the TCA cycle in mitochondria [19].

Through the characterization of a genetic mouse model of the cardiac-specific ablation of the carnitine palmitoyltransferase I (CPT I), researchers identified a critical role of CPT I in LCAC production and thereby the regulation of cardiac contractile function. In that study, it was shown that CPT I knockout led to significant left ventricular systolic dysfunction in response to a pressure overload [43,44]. Zhang and coworkers observed attenuated left ventricular hypertrophy and contractile dysfunction in a model of obesity-related cardiomyopathy after lentivirus-mediated CPT I suppression and an LCAC production blockade. By contrast, treatment of isolated Langendorff-perfused rodent hearts with LCACs induced pronounced contractile dysfunction [45], suggesting that excess LCAC levels, as seen in our in C18- and C18:1-treated zebrafish embryos, also interfere with regular cardiac function. Furthermore, it has been shown that treatment with the LCACs C14 and C16 led to the redistribution of Ca^2+^ from the sarcoplasmatic reticulum to mitochondria, resulting in a Ca^2+^ overload and thereby the shutdown of the TCA cycle, eventually leading to disturbed contractility in rat cardiomyocytes [21]. Whether disturbed calcium homeostasis also accounts for the defective ATP production and heart failure observed in our C18- and C18:1-treated zebrafish has to be investigated in future studies.

Our present study, using the zebrafish as a model system, connects the LCACs C18 and C18:1 and their impact on mitochondrial function to reduced cardiac contractile performance in vivo.

## 4. Materials and Methods

### 4.1. Animals and Imaging

Zebrafish husbandry was performed in accordance with institutional (Tierforschungszentrum (TFZ) Ulm University; No. 0183, 24.03.2011) and national (Germany) ethical and animal welfare regulation (Tierschutzgesetz § 11). The experimental procedures in this study were performed according to the guidelines from the EU Directive 2010/63/EU on the protection of animals used for scientific purposes. Zebrafish (*Danio rerio*) were bred and maintained at 28.5 °C as described by Westerfield [46]. Pictures and videos were taken at 96 h post-fertilization (hpf). To inhibit pigmentation, 0.003% 1-phenyl-2- thiourea was added to the regular embryo medium E3 (5 mM NaCl, 0.17 mM KCl, 0.33 mM CaCl_2_, 0.33 mM MgSO_4_ dissolved in water). Heart rate was counted at 96 hpf at room temperature (RT). Whole-mount zebrafish images were taken with an Olympus SZX 16 microscope (Olympus, Tokyo, Japan).

### 4.2. Acylcarnitine Preparation and Embryo Treatment

Acylcarnitines (C3: Sigma #20064-19-1; C18, C18:1: Advent Bio #52280, #43640) were dissolved in ddH_2_O (10 mg/mL, stock solution) by sonication and stored at −20 °C. For treatment, the corresponding LCAC concentration was obtained by diluting the stock solution with embryo medium E3. Zebrafish embryos at 48 hpf were incubated for 48 h at 28.5 °C with respective concentrations of C3, C18 and C18:1. The solution was renewed after 24 h of incubation.

### 4.3. Fractional Shortening

We measured fractional shortening by means of video microscopy (Leica, Wetzlar, Germany) at RT with open-source Image J software (U. S. National Institutes of Health, Bethesda, Maryland, USA; Available online: https://imagej.nih.gov/ij/, accessed on 5 July 2021). The diameters (short axis and long axis) of the ventricular lumen at the end of contraction (systole) and relaxation (diastole) were measured and calculated as described before [47].

### 4.4. MF20/S46 Immunostaining

For MF20/S46 immunostaining, embryos were collected at 96 hpf and fixed overnight in Dent′s fixative at RT, followed by a second incubation with Dent′s Bleach overnight at RT. Next, the embryos were transferred to 100% methanol. Slow rehydration of the embryos was performed at RT in a methanol row (20 min each 75%, 50% and 25% methanol in PBT) and in 0.1% Triton X-100 PBS (PBT). Blocking was carried out with 10% FCS in PBT for 2 h at RT; the embryos were incubated with primary antibody (MF20, 1:10) overnight. After washing 4 times for 30 min in blocking buffer, the embryos were incubated overnight with the next primary antibody (S46, 1:50). Subsequently, embryos were incubated overnight with both fluorescent secondary antibodies (Alexa 555 or 488, 1:100) protected from light. The next day, embryos were rinsed 3 times for 30 min in PBT. Finally, the embryos were visualized, and results were analyzed.

### 4.5. Counting Cardiomyocytes

Pictures of the fluorescent cardiomyocytes (CMs) were taken with a Leica DMi8 confocal microscope (Leica Microsystems, Wetzler, Germany). To count CMs on dissected hearts, the *Tg*(*myl7:dsRed.nuc*) line was used and the hearts were stained with dsRed (Takara Bio Inc., Kusatsu City, Shiga Prefecture, Japan) to enhance the fluorescence. Z-stack images were taken using the confocal microscope with a step size of 0.44 µm and a 40× oil objective. Counting CMs was performed with the ImageJ cell counter and the point tracker plugin. For statistics, the data were analyzed using GraphPad Prism9.

### 4.6. RNA Extraction and Quantitative Real-Time PCR

For biological replicates, a pool of 20 embryos was collected at 96 hpf. RNA extraction was carried out using an RNeasy Mini Kit (Qiagen, Düsseldorf, Germany) according to the manufacturer′s instructions. Total RNA (200 ng) was reverse transcribed to produce cDNA using Superscript III reverse transcriptase (Life Technologies, Carlsbad, CA, USA). Quantitative real-time PCR was carried out according to the standard protocols using SYBR Green (Roche, Basel, Switzerland) on a Roche Light Cycler 480 II. Two house-keeping genes, *β-actin* and *rpl13*, were used as reference genes for the normalization against other genes.

### 4.7. Histology

For histology, embryos were fixed with 4% paraformaldehyde and embedded in JB-4 (Polysciences, Inc, Philadelphia, PA, USA). Then, 5-µm sections were cut and dried, and samples were stained with hematoxylin and eosin. Transmission electron micrographs (TEMs) were obtained essentially as described previously [48]. Mitochondrial density and average size in TEM images were measured with Image J.

### 4.8. ATP Analysis

Sodium fluoroacetate (SFA) (FCH_2_CO_2_Na) was added at a concentration of 0.2 mg/mL to the medium of dechorionated wild-type zebrafish at 24 hpf and exchanged every 24 h. SFA blocks aconitate hydratase, thereby inhibiting the TCA cycle [49]. ATP content was measured with a luciferase-based assay (ATP Determination Kit A22066, Molecular probes, Invitrogen) with recombinant firefly luciferase and D-luciferin. Zebrafish embryos were mechanically lysed, and luminescence was immediately quantified using a microplate reader (TECAN). The ATP content was calculated by creating an ATP luminescence standard curve in Microsoft Excel.

### 4.9. Statistics

All results are depicted as the arithmetic mean ± standard deviation of the mean from at least three independent experiments. Statistical analyses were performed in GraphPad Prism9. Comparisons between experimental groups were performed using a *t*-test or two-way ANOVA. Differences were considered significant at the following *p*-values: *p* < 0.05, labeled “*”; *p* < 0.01, labeled “**”; *p* < 0.001, labeled “***”; and *p* < 0.0001, labeled “****”.

## 5. Conclusions

Based on our findings in the vertebrate in vivo model system of zebrafish, we demonstrated here that the excessive uptake of LCACs impairs mitochondrial ATP production, thereby leading to severe cardiac dysfunction. These findings suggest that orchestration and fine-tuning of LCAC levels in the heart is critical for regular mitochondrial activity and energy production to guarantee proper cardiac contractile function.

## Data Availability

The data presented in this study are available from the corresponding author.

## Supplementary Materials

The following are available online at https://www.mdpi.com/article/10.3390/ijms22168468/s1.
