# Supplementary material for: Long-Chain Acyl-Carnitines Interfere with Mitochondrial ATP Production Leading to Cardiac Dysfunction in Zebrafish
Source: Int J Mol Sci. 2021 Aug 6;22(16):8468. doi: 10.3390/ijms22168468 (PMC8395116; doi:10.3390/ijms22168468)
Supplement: Supplementary file 1 [file ijms-22-08468-s001.zip › ijms-1323149-supplementary.pdf]

Supplementary Table S1

Supplementary Table S1. Sequence of primers used for qRT-PCR

	Name	Sequence
qRT-PCR	zmfn1a_qRT_for	TGTGGAGGAGAGAATGGTGA
	zmfn1a_qRT_rev	GAGCAGAGGAAGAGGTGAGC
	zmfn1b_qRT_for	CTGCCGAACCCGAATACAT
	zmfn1b_qRT_rev	GCGATCCACCACCTTCAG
	zpparg_qRT_for	AGCTACAGCCCTGAGGAGAA
	zpparg_qRT_rev	GAGGAGATTCGGGCTCAAG
	zppargc1a_qRT_for	CCTGCTAACTCCCAGCTCAG
	zppargc1a_qRT_rev	GGGGTTTTCTGTCTTGGCAAC
	zcox4i1_qRT_for	GGTCGGAGACGCTAGAATGT
	zcox4i1_qRT_rev	AGTAGTCCTCGACCTTCGCA
	zcox4i2_qRT_for	AACCAACCGGTGAGTGGAAG
	zcox4i2_qRT_rev	TGAGGAGGATACACATAGAGCCT
	zrpl13_qRT_for	TCTGGAGGACTGTAAGAGGTATGC
	zrpl13_qRT_rev	AGACGCACAATCTTGAGAGCAG

Supplementary Figure S1

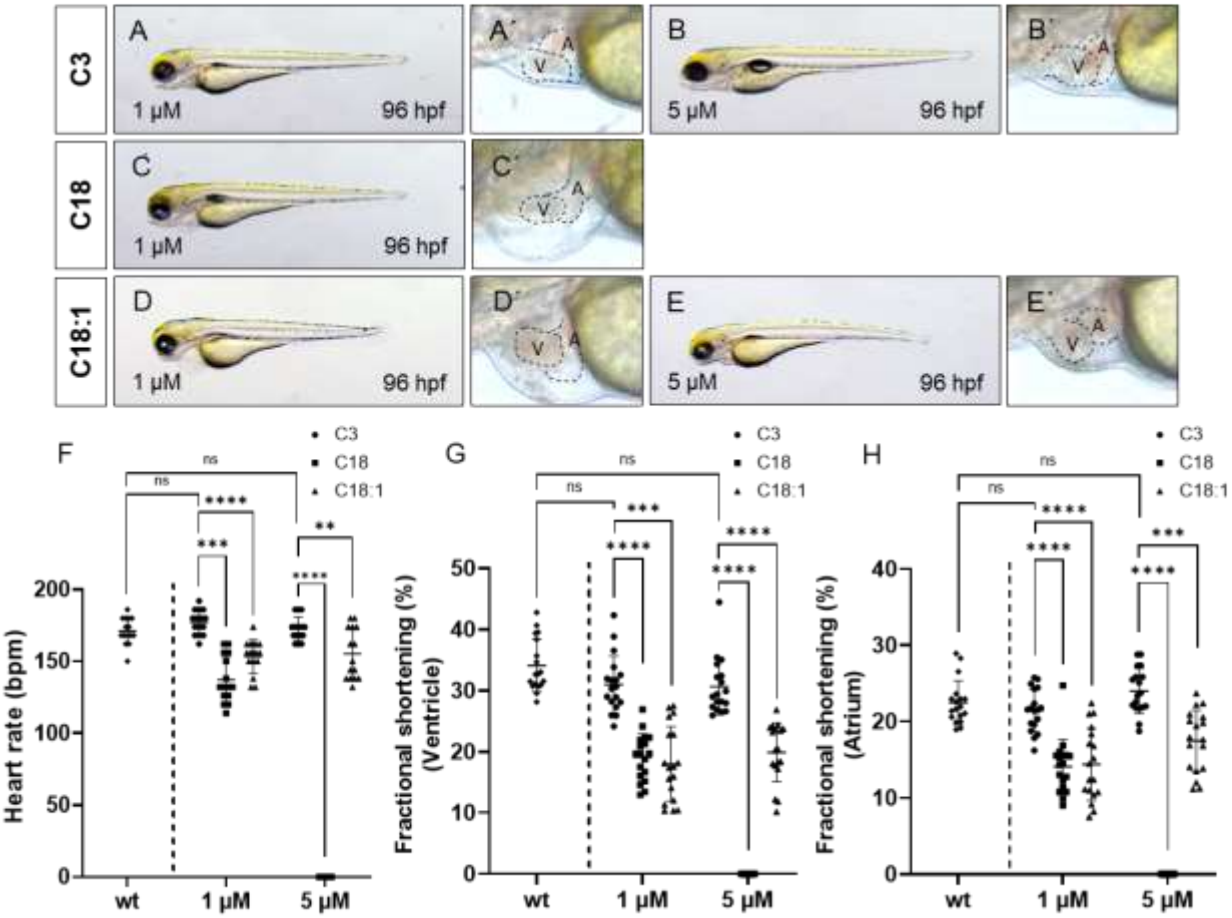

Supplementary Figure S1. Phenotype and cardiac function of zebrafish embryos under higher concentration of LCACs. (A-E) Lateral view of embryos after compounds incubation. (A´-E´) Magnified view of hearts from B-G. (F) Comparison of heart rate between wild-type and carnitines treated larvae (wt: 171.2 ± 9.31, bpm ; C3: 1 µM: 177.2 ± 8.45, 5 µM: 172.4 ± 8.32; C18: 1 µM: 137.2 ± 16.01, 0.5 µM: 0; C18:1: 1 µM: 153.2 ± 11.76, 0.5 µM: 155.2 ± 17.69, bpm, SD, n=15, $p < 0.01$, $p < 0.001$, $p < 0.0001$) (G, H) Fractional shortening (FS) of heart chambers after compound incubation compared to wt (Ventricular FS; wt: 33.52 ± 4.22%; C3: 1 µM: 30.98 ± 4.66%, 5 µM: 30.05 ± 4.58%; C18: 1 µM: 19.10 ± 3.82%, 5 µM: 0%; C18:1: 1 µM: 17.99 ± 6.09%, 5 µM: 19.88 ± 4.82%, SD, n=18, ns: $p > 0.05$, $p < 0.001$, $p < 0.0001$) (Atrial FS; wt: 22.46 ± 2.88%; C3: 1 µM: 21.46 ± 2.77%, 5 µM: 24.01 ± 2.97%; C18: 1 µM: 14.09 ± 3.55%, 5 µM: 0%; C18:1: 1 µM: 14.37 ± 4.73%, 0.5 µM: 17.46 ± 4.01%, SD, n=18, ns: $p > 0.05$, $p < 0.001$, $p < 0.0001$).

Supplementary Figure S2

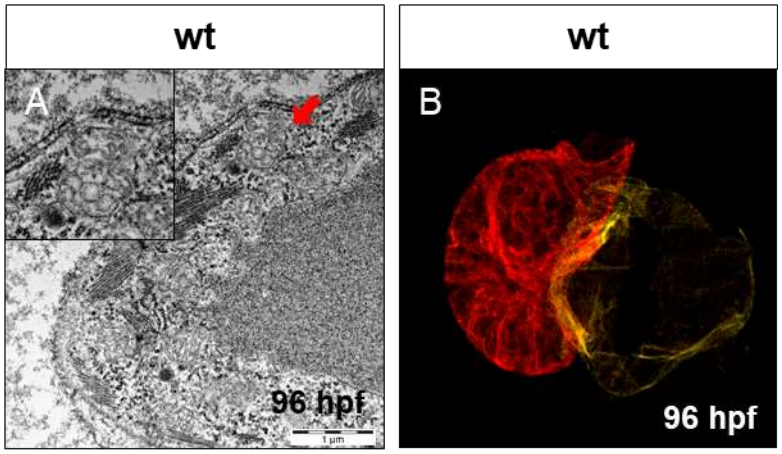

Supplementary Figure S2. Electron microscope image (A) and MF20/S46 staining (B) of wild-type embryonic zebrafish heart.
